# The Indirect Influence of Stroke Performances on Point Scoring and Conceding in the Four Primary Table Tennis Match Formats

**DOI:** 10.5114/jhk/196651

**Published:** 2025-10-01

**Authors:** Jiangchuan Yu, Zhifeng Huang

**Affiliations:** 1 Sports Coaching College, Beijing Sport University, Beijing, China.; 2 Key Laboratory of Sport Training of General Administration of Sport of China, Beijing Sport University, Beijing, China.

**Keywords:** indirect influence, stroke techniques, ball placement, point dynamics, dominant hand

## Abstract

The indirect impact of stroke performance on the dynamics of winning and losing points in table tennis has been seldom explored. This study aimed to uncover and contrast the nuances of stroke execution—both the techniques utilized and the strategic ball placements—in four principal types of table tennis matches: right-handed versus right-handed (RRM), right-handed versus left-handed (RLM), left-handed versus right-handed (LRM) and left-handed versus left-handed (LLM). These matches exhibit distinct indirect effects on rally outcomes, categorized as positive (winner-assisting) and negative (momentum-diminishing). Our analysis encompassed 190 men's singles matches with a total of 15916 points played. Employing chi-square tests for multiple bivariate analyses and subsequent post-hoc pairwise comparisons, we found that for the development of winner-assisting strokes, the ball placements from the normal sidespin serve, backspin/no-spin serve, short push, long push, forehand flip, backhand flip, and both non-topspin and topspin loop/drives for both the forehand and the backhand had small to large effects (p < 0.05–0.001, ES: 0.084–0.266). Similarly, for the induction of momentum-diminishing strokes, the ball placements from the aforementioned serves, short/long push, and flips, along with the non-topspin and topspin loop/drives, blocks and lobs, yielded small to moderate effects (p < 0.05–0.001, ES: 0.050–0.206). These insights are invaluable, enabling professionals to grasp table tennis strategies, enhance training, and execute match plans with precision.

## Introduction

Table tennis, renowned for its blend of skill and strategy, enjoys global popularity (Xiao et al., 2018). Scholars have long been captivated by research into the technical-tactical performance in elite table tennis matches, seeking to uncover the pivotal determinants of victory. This interest has yielded a steady stream of research findings in recent years (Djokic et al., 2020; Liu and Lames, 2024; Pradas et al., 2023; Wang et al., 2022). From the perspective of the practical application of the results, studies with the topic of mining both successful and unsuccessful patterns of play in table tennis matches can provide valuable sources and references for efficient training and targeted match-strategy formulation to professionals at the operational level (Yu, 2023).

Table tennis performance analysis hinges on the rally, a sequence of alternating strokes that players execute (Wenninger and Lames, 2016; Xiao et al., 2018). The server (on odd shots) and the receiver (on even shots) use different stroke actions to force the opponent to make mistakes in returning the ball, thereby achieving victory (Guo et al., 2020; Wang, 2019). Typically, two categories of variables are essential for conducting a notational analysis of players' performance. One category includes the technical-tactical performance indicators. The other encompasses the indices that are used to assess them.

The primary performance indicators used for analysis include stroke technique (Djokic et al., 2020; Munivrana et al., 2015; Wang, 2019; Yu, 2023), ball placement (Djokic et al., 2020; Munivrana et al., 2015; Wang, 2019; Zhang and Zhou, 2017), ball trajectory (Guo et al., 2020; Pradas de la Fuente et al., 2023) and the stroke position (Pfeiffer et al., 2010; Wang, 2019), as they are both recordable and capable of precisely reflecting players' technical-tactical behaviors. Among these variables, the stroke position when performing a shot is totally depended on the ball placement of the opponent’s last stroke. Moreover, the ball trajectory is the connecting line between the ball placements of the opponent’s last stroke and the player’s current stroke, which is also partly affected by the opponent. However, the employment of the stroke technique and the ball placement are entirely determined by the performer, and these indicate the initiative of the performing player.

For evaluating performance indicators, numerous studies have assessed their effectiveness based on the direct influence on rally outcomes. Common evaluation indices include the score difference (Tamaki et al., 2017), the scoring rate and the losing rate (Guo et al., 2020; Xiao et al., 2018; Zhang et al., 2018) as well as the technical effectiveness (Pfeiffer et al., 2010; Tamaki et al., 2017; Wang, 2019; Zhang et al., 2013). These indices were designed to capture the immediate effects of technical-tactical actions during rallies, with the goal of uncovering the most impactful outcomes of each evaluated metric. Yet, in table tennis matches, scoring or conceding a point is not solely contingent upon the final shot exchange of a rally, the performance of preceding strokes also holds significant utility (Yu and Gao, 2022). Therefore, in order to comprehensively examine the influence of performance indicators, the indirect role that they played should not be neglected to accomplish a thorough assessment.

Previous studies typically categorized the function of a stroke into three types, i.e., a winner (a scoring shot), a return (a transition shot) and an error (a missing shot) (Malagoli et al., 2014; Tamaki et al., 2017). Zhang et al. (2020) further classified the effect of a stroke into a direct score, an indirect score, a transition, an indirect loss and a direct loss. The “indirect-scoring stroke” refers to the antepenultimate shot in a rally, which is also the shot immediately preceding the decisive stroke that secures the point for the scoring side. On the other hand, the “indirect-losing stroke” is the penultimate shot in a rally, which is the last successful return performed by the losing player. Since there is a direct correlation between the adjacent strokes (Jiang and Yao, 2015), the performances of the two strokes would indirectly lead to the termination of a rally, resulting in a score and a loss. However, according to the literature review, previous studies had not systematically investigated the performance of the two strokes. Therefore, our study intended to fill this gap and expand professionals' understandings of the table tennis match performances from a new perspective.

Additionally, in interactive sports, the dominant hand of an athlete, whether left or right, can significantly shape their performance. Empirical comparative studies have consistently demonstrated that left-handed athletes exhibit distinct technical-tactical profiles compared to their right-handed counterparts. For instance, in table tennis, Malagoli Lanzoni et al. (2019) delved into the potential strategic advantages afforded to left-handed players, highlighting their increased adaptability and aggressiveness in both serving and receiving strategies. In the realm of badminton, Zhang and Leng (2024) identified significant differences in technique and tactical utilization between left-handed and right-handed players, suggesting these disparities could influence the game's tempo and the ultimate outcome. Within the domain of tennis, a series of studies by Loffing et al. (2009, 2010, 2012) brought to light the distinctive serving styles, shot angles, and tactical decisions of left-handed players, which were posited to confer a strategic edge. Furthermore, in volleyball, research such as the experimental work by Loffing et al. (2015) demonstrated that left-handed players exhibited marked differences from their right-handed counterparts in the strategic decision-making processes during blocking and attacking phases. These insights underscore the critical role of dominant hand variation in shaping an athlete's approach to training and competition, and they stress the importance of conducting tailored analyses of matches that account for the different dominant hand preferences of the athletes involved.

Thus, this paper's research objective was to analyze and compare the stroke performances—specifically, stroke technique and ball placement—responsible for scoring (the antepenultimate stroke, known as the winner-assisting stroke) and conceding points (the penultimate stroke, referred to as the momentum-diminishing stroke) across the four formats of elite men's table tennis competitions characterized by the handedness dynamics between the two competing players. The results of the present study could provide insights for professionals, enabling them to optimize training plans and to refine match strategies when competing in different match forms.

## Methods

### Sample

The research included an extensive dataset, comprising 190 men's singles table tennis matches, which accounted for 15916 individual points. Matches were sourced from prestigious table tennis events: the Olympic Games, World Championships, World Cup, ITTF World Tour and World Tour Grand Finals, WTT Champions and WTT Grand Smash spanning the period from 2018 to 2024. Moreover, the sampled matches were categorized into four distinct types based on the handedness of the competitors: right-handed versus right-handed (RRM), right-handed versus left-handed (RLM), left-handed versus right-handed (LRM), and left-handed versus left-handed (LLM). The handedness of players was established according to which hand was used to hold the racket (Peters and Murphy, 1992). For RLM and LRM formats, players’ performance was analyzed separately for each side according to the handedness indicated by the initial letter. In contrast, for RRM and LLM formats, performance of players on both sides was analyzed collectively.

Out of the total, 82 matches were between right-handed players (RRM), involving 37 players with an average age of 27.4 years, a standard deviation of 5.0 years, and world rankings spanning from 1 to 47. Additionally, there were 78 matches for both right-handed versus left-handed (RLM) and left-handed versus right-handed (LRM) players, encompassing 29 competitors with an average age of 26.6 years, a standard deviation of 5.4 years, and world rankings from 1 to 65. The study also encompassed 30 matches featuring left-handed players (LLM), with 18 athletes averaging 25.9 years in age, a standard deviation of 6.1 years, and world rankings ranging between 1 and 50.

Exclusion criteria for the data were points from rallies that ended with service faults or reception errors, as well as points that were not observable due to recording problems. As a result, the dataset for analyzing the momentum-diminishing stroke (the second-to-last stroke of a rally) included 7212 points for RRM, 3619 for RLM, 2858 for LRM, and 2227 for LLM formats. Additionally, the total number of points analyzed for the winner-assisting stroke (the third-to-last stroke of a rally) was 5873 for RRM, 2855 for RLM, 2204 for LRM, and 1828 for LLM formats. Among these, 1339, 764, 654, and 399 points ended with a serve-return winner and a third-stroke error for RRM, RLM, LRM, and LLM formats, respectively. The study protocol was in compliance with the ethical standards of the Declaration of Helsinki.

### Performance Indicators

The study utilized the stroke technique and ball placement of both the winner-assisting and momentum-diminishing strokes as key performance indicators. In terms of stroke technique classification, this study drew upon prior research to categorize techniques into thirteen distinct types (Wang et al., 2019; Yu, 2023; Zhang and Zhou, 2017). Notably, techniques with analogous impacts, such as a loop and a drive, and a block and a lob, were consolidated into single categories. Furthermore, the primary attacking strokes—loops and drives—were differentiated into two subtypes based on their function: one for returning non-topspin balls and another for countering topspin balls (Yu and Gao, 2022). The classifications and operational definitions of the stroke techniques are detailed in Table 1.

**Table 1:** Classifications and operational definitions of stroke technique.

Stroke technique	Operational definition
Normal sidespin (T/B) serve	Player serves by an outward and inclined racket-face angle to hit the ball at the side close to the torso to produce a deflected ball trajectory towards the lateral side of the body.
Reverse sidespin (T/B) serve	Player serves by an inward and inclined racket-face angle to hit the ball at the side away from the torso to produce a deflected ball trajectory towards the lateral side of the body.
Backspin/no-spin serve	Player serves by an upward and inclined racket-face angle to hit the ball at the lower half of the ball to produce a straight ball trajectory.
Short push	Player performs a controlling stroke over the table by an upward and inclined racket-face angle to hit the ball at the lower half to produce a backspin return with a placement near the net.
Long push	Player performs a controlling stroke over the table by an upward and inclined racket-face angle to hit the ball at the lower half to produce a backspin return with a placement close to the end line.
Forehand Flip	Player performs an attacking stroke over the table using the forehand-side rubber by a vertical or a downward racket-face angle to hit the ball at the middle or the upper half to produce a topspin return with a placement close to the end line.
Backhand Flip	Player performs an attacking stroke over the table using the backhand-side rubber by a vertical or a downward racket-face angle to hit the ball at the middle or the upper half to produce a topspin or side-topspin return with a placement close to the end line.
Forehand loop/drive (NT)	Player performs an attacking stroke outside the end line of the table using the forehand-side rubber by a vertical or a downward racket-face angle to hit the ball at the middle or the upper half to attack a non-topspin ball and produce a topspin return with a placement close to the end line.
Backhand loop/drive (NT)	Player performs an attacking stroke outside the end line of the table using the backhand-side rubber by a vertical or a downward racket-face angle to hit the ball at the middle or the upper half to attack a non-topspin ball and produce a topspin return with a placement close to the end line.
Forehand loop/drive (T)	Player performs an attacking stroke outside the end line of the table using the forehand-side rubber by a vertical or a downward racket-face angle to hit the ball at the middle or the upper half to counter a topspin ball and produce a topspin return with a placement close to the end line.
Backhand loop/drive (T)	Player performs an attacking stroke outside the end line of the table using the backhand-side rubber by a vertical or a downward racket-face angle to hit the ball at the middle or the upper half to counter a topspin ball and produce a topspin return with a placement close to the end line.
Forehand block/lob	Player performs a defensive stroke outside the end line of the table using the forehand-side rubber by an upward, a vertical or a downward racket-face angle to hit the ball at the lower half, the middle or the upper half to ricochet a topspin ball and produce a topspin or a no-spin return with a placement close to the end line.
Backhand block/lob	Player performs a defensive stroke outside the end line of the table using the backhand-side rubber by an upward, a vertical or a downward racket-face angle to hit the ball at the lower half, the middle or the upper half to ricochet a topspin ball and produce a topspin or a no-spin return with a placement close to the end line.

Note: (T/B) refers to the topspin or the backspin; (NT) refers to the non-topspin; (T) refers to the topspin

**Figure 1:**
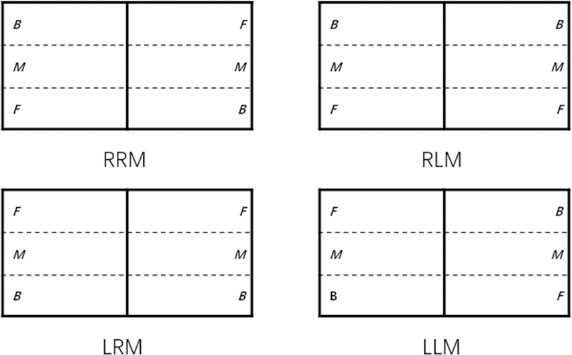
Zone distribution of ball placement of the four match formats. Note: RRM: a right-handed player against a right-handed player match, RLM: a right-handed player against a left-handed player match. LRM: a left-handed player against a right-handed player match, LLM: a left-handed player against a left-handed player match. F: forehand zone; M: middle zone; B: backhand zone.

Regarding ball placement categorization, the nine-or-six-area division method is commonly employed (Malagoli et al., 2014; Wang, 2019; Zhang et al., 2010). However, this study adopted a three-zone partition approach, as the stroke technique employed can reflect the vertical dimension of the ball's landing location on the table (Guo et al., 2020; Wang, 2019; Yu, 2023). The distributions of ball placements for both match formats are illustrated in Figure 1.

### Data Collection and Reliability

Two seasoned table tennis analysts were engaged for data collection. Prior to commencing match observation, a comprehensive briefing on the study's variables was provided, achieving consensus between the analysts. To assess data reliability, ten matches from each format were randomly selected for analysis. The Kappa coefficient was utilized to measure the level of concordance among the notational data (Altman, 1990).

Within the RRM format, the intra-observer reliability for the winner-assisting and momentum-diminishing strokes achieved scores of 0.98 and 0.97, respectively. The inter-observer reliability for these strokes were 0.96 and 0.95, respectively. In the RLM format, the intra-observer reliability scores for the winner-assisting and momentum-diminishing strokes were 0.96 and 0.95, while the inter-observer reliability scores were 0.95 and 0.93, respectively. For the LRM format, both intra-observer reliability scores for the winner-assisting and momentum-diminishing strokes were 0.97, and the inter-observer reliability scores were 0.95 for both types of strokes. In the LLM format, the intra-observer reliability scores were 0.96 for the winner-assisting stroke and 0.94 for the momentum-diminishing stroke, with inter-observer reliability scores of 0.93 and 0.92, respectively. Collectively, these results indicate a very high degree of consistency across all tests, affirming the reliability of the observational data (Donoghue, 2009).

### Statistical Analysis

Bivariate analysis using chi-square (χ^2^) tests was performed to evaluate the correlations between the use of ball placements with each stroke technique in both the winner-assisting and momentum-diminishing strokes and the four match formats. The statistical significance threshold was set at *p* < 0.05. Cramer's V coefficient was employed to quantify the strength of associations, with correlations classified as small (V < 0.1), moderate (0.1 ≤ V < 0.25), large (0.25 ≤ V < 0.4) and very large (V ≥ 0.4) based on Kline 's (2013) criteria. Following this, post-hoc pairwise comparisons were carried out to identify which specific ball placements for each stroke technique correlated with the match format, as suggested by the initial analysis. The Bonferroni correction adjusted the significance level from 0.05 by dividing it by the number of comparisons conducted. With eighteen comparisons per stroke technique, the adjusted significance level was *p'* < 0.0028. The phi coefficient (φ) was used to measure effect size. All statistical analyses were conducted using IBM SPSS Statistics, version 27.0.1.

## Results

### Winner-Assisting Stroke

The application of a range of stroke techniques, such as the shot push (χ^2^ = 28.949, *p* < 0.001), the normal sidespin (in addition to the topspin and the backspin) serve (χ^2^ = 39.672, *p* < 0.001), the long push (χ^2^ = 13.579, *p* < 0.05), the forehand loop/drive against topspin balls (χ^2^ = 37.028, *p* < 0.001), the backhand flip (χ^2^ = 25.903, *p* < 0.001), the backspin/no-spin serve (χ^2^ = 29.648, *p* < 0.001), the forehand loop/drive against non-topspin balls (χ^2^=23.783, *p* < 0.001), the backhand loop/drive against topspin balls (χ^2^ = 229.682, *p* < 0.001), the forehand flip (χ^2^ = 23.303, *p* < 0.001) and backhand loop/drive against non-topspin balls (χ^2^ = 52.485, *p* < 0.001) were significantly associated with the generation of winning shots across four distinct match formats. These techniques yielded an influence that ranged from small to large, with associated effect sizes from 0.083 to 0.266 (Table 2).

**Table 2:** Multiple comparisons between performance variables and match formats considering the winner-assisting stroke.

ST	BP	RRM	RLM	LRM	LLM	χ^2^	Sig.	Cramer’s V
NSS	F	288(23.5)	192(30.6)	143(25.7)	143(35.3)	39.672	<0.001	0.084
	M	745(60.7)	323(51.5)	327(58.8)	182(44.9)			
	B	195(15.9)	112(17.9)	86(15.5)	80(19.8)			
RSS	F	112(37.8)	55(35.9)	50(40.0)	57(45.6)	8.928	0.178	0.079
	M	122(41.2)	72(47.1)	68(48.9)	42(33.6)			
	B	62(20.9)	26(17.0)	21(15.1)	26(20.8)			
BNSS	F	68(28.0)	62(40.3)	38(36.5)	23(44.2)	29.648	<0.001	0.164
	M	81(33.3)	66(42.9)	45(43.3)	14(26.9)			
	B	94(38.7)	26(16.9)	21(20.2)	15(28.9)			
SP	F	317(29.2)	147(30.2)	93(31.8)	72(29.1)	28.949	<0.001	0.083
	M	686(63.3)	300(61.6)	185(63.4)	134(54.3)			
	B	81(7.5)	40(8.2)	14(4.8)	41(16.6)			
LP	F	25(8.0)	15(11.4)	12(11.2)	11(13.4)	13.579	<0.05	0.103
	M	127(40.6)	35(26.5)	45(42.1)	23(28.1)			
	B	161(51.4)	82(62.1)	50(46.7)	48(58.5)			
FF	F	22(25.9)	6(12.2)	4(9.7)	12(40.0)	23.303	<0.001	0.238
	M	29(34.1)	15(30.6)	10(24.4)	13(43.3)			
	B	34(40.0)	28(57.2)	27(65.9)	5(16.7)			
BF	F	29(9.3)	30(15.9)	28(21.4)	10(8.8)	25.903	<0.001	0.132
	M	113(36.2)	81(42.9)	59(45.0)	50(43.8)			
	B	170(54.5)	78(41.3)	44(33.6)	54(47.4)			
FLDN	F	47(24.1)	11(10.9)	10(11.2)	11(24.4)	23.783	<0.001	0.166
	M	65(33.3)	21(20.8)	25(28.1)	14(31.1)			
	B	83(42.6)	69(68.3)	54(60.7)	20(44.4)			
BLDN	F	8(4.8)	25(39.1)	15(24.2)	6(7.5)	52.485	<0.001	0.266
	M	65(39.2)	17(26.6)	21(33.9)	27(33.8)			
	B	93(56.0)	22(34.4)	26(41.9)	47(58.7)			
FLDT	F	180(25.0)	82(18.9)	49(16.3)	67(27.9)	37.028	<0.001	0.105
	M	266(36.9)	129(29.7)	91(30.2)	66(27.5)			
	B	274(38.1)	223(51.4)	161(53.5)	107(44.6)			
BLDT	F	89(8.4)	151(35.8)	111(34.8)	31(11.9)	229.682	<0.001	0.236
	M	454(43.0)	150(35.5)	123(38.6)	114(43.7)			
	B	512(48.5)	121(28.7)	85(26.6)	116(44.4)			
FBL	F	13(19.1)	5(16.1)	4(23.5)	1(6.3)	8.217	0.223	0.176
	M	36(52.9)	18(58.1)	4(23.5)	8(50.0)			
	B	19(27.9)	8(25.8)	9(53.0)	7(43.7)			
BBL	F	15(13.9)	14(24.6)	13(26.0)	7(17.1)	8.537	0.201	0.129
	M	53(49.1)	32(56.1)	24(48.0)	22(53.6)			
	B	40(37.0)	11(19.3)	13(26.0)	12(29.3)			

Note: The numbers and percentages in the matching parentheses below RRM, RLM, LRM and LLM are the frequencies and relative frequencies of the corresponding stroke technique and ball placement. ST: stroke technique; BP: ball placement; RRM: righthanded against righthanded match; RLM: righthanded against lefthanded match; LRM: lefthanded against righthanded match; LLM: lefthanded against lefthanded match; NSS: normal sidespin (in addition to the topspin or the backspin) serve; RSS: reverse sidespin (in addition to the topspin or the backspin) serve; BNSS: backspin or no-spin serve; SP: short push; LP: long push; FF: forehand flip; BF: backhand flip ; FLDN: forehand loop or drive against non-topspin balls; BLDN: backhand loop or drive against non-topspin balls; FLDT: forehand loop or drive against topspin balls; BLDT: backhand loop or drive against topspin balls; FBL: forehand block or lop; BBL: backhand block or lop; F: forehand zone; M: middle zone; B: backhand zone

**Figure 2:**
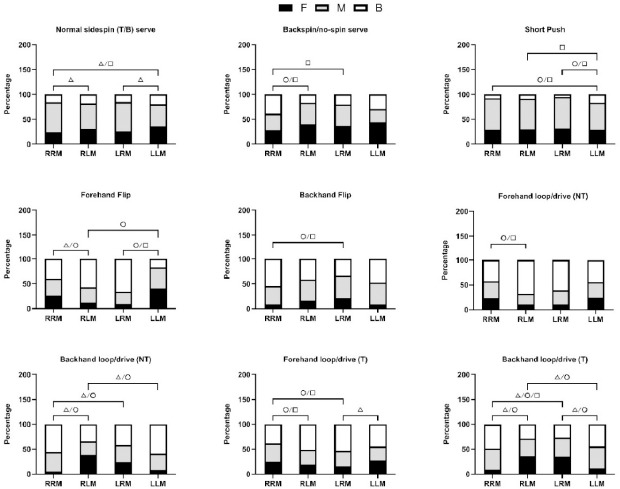
Post-hoc pairwise comparisons of the stroke techniques and the ball placements that had significant relationships with the match format for the winner-assisting stroke. Note: Significance level was p' < 0.0028. F: forehand zone; M: middle zone; B: backhand zone △: significant difference between F and M; ○: significant difference between F and B; □: significant difference between M and B

Figure 2 displays the results of post-hoc pairwise comparisons. When comparing RRM to RLM formats, the normal sidespin serve exhibited a marked preference for the middle zone in the RRM format (60.7%), in contrast to RLM's preference for the forehand zone (30.6%). The backspin or no-spin serve was notably more frequent in the forehand zone in the RRM format (28.0%) and the backhand zone (38.7%) compared to the RLM format. In the comparison between RRM and LRM formats, the normal sidespin serve's middle zone preference in the RRM format (60.7%) was significantly higher than in the LRM format, which favored the forehand zone (25.7%). The backhand loop and drive against non-topspin balls showed a pronounced difference, with the RRM format favoring the forehand zone (4.8%) and the middle zone (39.2%) over the LRM format.

When contrasting the RRM with the LLM format, the middle zone was again the preferred target for the normal sidespin serve in the RRM format (60.7%), whereas the LLM format saw a greater inclination towards the forehand zone (35.3%). The short push was considerably less common in the backhand zone in the RRM (7.5%) than in the LLM format (16.6%). Additionally, both the forehand flip and the backhand flip displayed significant variations in the forehand zone usage between RRM (25.9% and 9.3%, respectively) and LLM formats (40.0% and 8.8%, respectively). When pitting the RLM against the LRM format, the backspin and no-spin serve's backhand zone usage in the RLM format (16.9%) was significantly higher than in the LRM format, which showed a greater preference for the middle zone (43.3%).

When juxtaposing RLM and LLM formats, the forehand flip's backhand zone usage in the RLM format (57.2%) was markedly higher than in the LLM format, which favored the forehand zone (40.0%). In the examination of the LRM versus the LLM format, the short push's backhand zone usage in the LRM format (4.8%) was significantly lower than in the LLM format (16.6%). Similarly, the backhand loop and drive against topspin balls' backhand zone usage in the LRM format (26.6%) was considerably less than in the LLM format (44.4%).

### Momentum-Diminishing Stroke

The deliberate ball placement during strokes, including the short push (χ^2^ = 13.061, *p* < 0.05), the long push (χ^2^ = 16.902, *p* < 0.001), the forehand loop/drive against topspin balls (χ^2^ = 41.891, *p* < 0.001), the backhand flip (χ^2^ = 23.239, *p* < 0.001), the backhand block or lob (χ^2^ = 21.272, *p* < 0.05), the forehand block or lob (χ^2^ = 12.798, *p* < 0.05), the backspin/no-spin serve (χ^2^ = 16.808, *p* < 0.05), the backhand loop/drive against non-topspin balls (χ^2^ = 27.466, *p* < 0.001), the forehand loop/drive against non-topspin balls (χ^2^ = 66.428, *p* < 0.001), the backhand loop/drive against topspin balls (χ^2^ = 231.475, *p* < 0.001) and the forehand flip (χ^2^ = 47.406, *p* < 0.001), were correlated with the indirect concession of points across the four match formats. These techniques, though individually impactful, collectively contributed to small to moderate effects, with effect sizes varying from 0.050 to 0.206 (Table 3).

**Table 3:** Multiple comparisons between performance variables and match formats considering the momentum-diminishing stroke.

ST	BP	RRM	RLM	LRM	LLM	χ^2^	Sig.	Cramer’s V
NSS	F	219(24.8)	169(31.3)	129(27.8)	74(28.2)	8.403	0.210	0.044
	M	489(55.4)	268(49.6)	236(50.9)	137((52.3)			
	B	175(19.8)	103(19.1)	99(21.3)	51(19.5)			
RSS	F	128(44.6)	58(42.3)	41(45.0)	38(42.7)	11.762	0.068	0.099
	M	116(40.4)	62(45.3)	32(35.2)	27(30.3)			
	B	43(15.0)	17(12.4)	18(19.8)	24(27.0)			
BNSS	F	33(19.5)	40(38.5)	36(36.4)	12(34.3)	16.808	<0.05	0.144
	M	69(40.8)	35(33.7)	39(39.4)	12(34.3)			
	B	67(39.6)	29(27.9)	24(24.2)	11(31.4)			
SP	F	375(30.3)	196(33.9)	160(35.3)	93(27.9)	13.061	<0.05	0.050
	M	759(61.3)	331(57.2)	251(55.4)	197(59.2)			
	B	105(8.5)	52(8.9)	42(9.3)	43(12.9)			
LP	F	57(9.4)	47(15.9)	33(16.5)	23(15.0)	16.902	<0.001	0.082
	M	257(42.4)	101(34.2)	82(41.0)	52(34.0)			
	B	292(48.2)	147(49.8)	85(42.5)	78(51.0)			
FF	F	51(35.9)	6(10.2)	6(9.1)	19(44.2)	47.406	<0.001	0.277
	M	55(38.7)	20(33.9)	29(43.9)	20(46.5)			
	B	36(25.4)	33(55.9)	31(47.0)	4(9.3)			
BF	F	54(12.3)	53(19.7)	33(15.2)	21(12.4)	23.239	<0.001	0.103
	M	136(30.9)	101(37.5)	90(41.5)	52(30.8)			
	B	250(56.8)	115(42.8)	94(43.3)	96(56.8)			
FLDN	F	123(31.5)	26(14.2)	23(16.8)	34(41.0)	66.428	<0.001	0.205
	M	131(33.6)	43(23.5)	28(20.4)	17(20.5)			
	B	136(34.9)	114(62.3)	86(62.8)	32(38.5)			
BLDN	F	36(12.5)	30(24.6)	30(28.3)	10(9.0)	27.446	<0.001	0.148
	M	99(34.5)	42(34.4)	27(25.5)	33(29.7)			
	B	152(53.0)	50(41.0)	49(46.2)	68(61.3)			
FLDT	F	261(27.4)	110(18.4)	87(22.8)	100(31.2)	41.891	<0.001	0.096
	M	386(40.6)	222(37.0)	133(34.9)	117(36.4)			
	B	304(32.0)	267(44.6)	161(42.3)	104(32.4)			
BLDT	F	126(9.1)	172(30.8)	125(30.6)	36(9.6)	231.475	<0.001	0.206
	M	611(44.1)	228(40.8)	167(40.8)	147(39.2)			
	B	648(46.8)	159(28.4)	117(28.6)	192(51.2)			
FBL	F	24(15.9)	11(15.4)	11(14.5)	16(29.1)	12.798	<0.05	0.135
	M	93(61.6)	45(63.4)	37(48.7)	27(49.1)			
	B	34(22.5)	15(21.1)	28(36.8)	12(21.8)			
BBL	F	43(15.2)	48(28.6)	22(13.9)	20(16.0)	21.272	<0.05	0.120
	M	152(53.9)	80(47.6)	93(58.9)	58(46.4)			
	B	87(30.9)	40(23.8)	43(27.2)	47(37.6)			

Note: The numbers and percentages in the matching parentheses below RRM, RLM, LRM and LLM are the frequencies and relative frequencies of the corresponding stroke technique and ball placement. ST: stroke technique; BP: ball placement; RRM: righthanded against righthanded match; RLM: righthanded against lefthanded match; LRM: lefthanded against righthanded match; LLM: lefthanded against lefthanded match; NSS: normal sidespin (in addition to the topspin or the backspin) serve; RSS: reverse sidespin (in addition to the topspin or the backspin) serve; BNSS: backspin or no-spin serve; SP: short push; LP: long push; FF: forehand flip; BF: backhand flip ; FLDN: forehand loop or drive against non-topspin balls; BLDN: backhand loop or drive against non-topspin balls; FLDT: forehand loop or drive against topspin balls; BLDT: backhand loop or drive against topspin balls; FBL: forehand block or lop; BBL: backhand block or lop; F: forehand zone; M: middle zone; B: backhand zone

**Figure 3:**
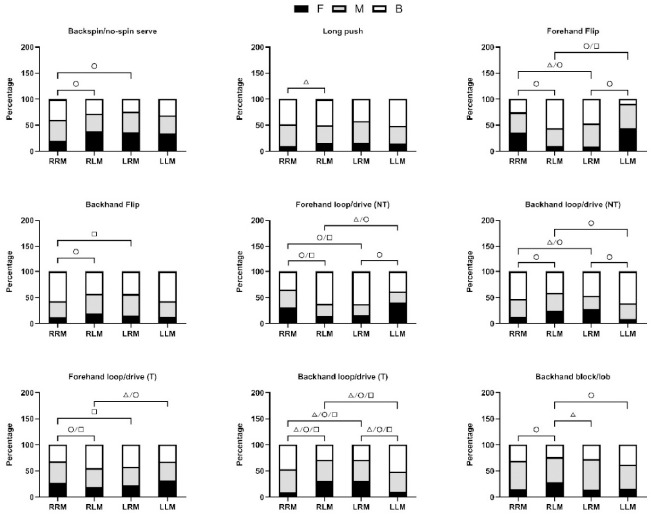
Post-hoc pairwise comparisons of the stroke techniques and the ball placements that had significant relationships with the match format for the momentum-diminishing stroke. Note: Significance level was p < 0.0028; F: forehand zone; M: middle zone; B: backhand zone △: significant difference between F and M; ○: significant difference between F and B; □: significant difference between M and B

Figure 3 shows the outcomes of post-hoc pairwise comparisons. When analyzing the differences between RRM and RLM formats, the forehand flip was markedly more frequent in the forehand zone in the RRM (35.9%) compared to the RLM format (10.2%). It was also observed that the backhand flip was significantly more prevalent in the backhand zone in the RRM (56.8%) than in the RLM format (42.8%). Additionally, the forehand loop/drive against non-topspin balls demonstrated significant variations, with RRM players targeting the forehand zone (31.5%) and the backhand zone (34.9%) more frequently than RLM players, who showed figures of 14.2% and 62.3%, respectively.

In the comparison of the RRM to the LRM format, the long push was notably more common in the forehand zone in the LRM (16.5%) than in the RRM format (9.4%). The backhand loop/drive against non-topspin balls also presented significant differences, with RRM players favoring the forehand zone (12.5%) and the backhand zone (53.0%), in contrast to LRM players' preferences at 28.3% and 46.2%. When contrasting the RRM with the LLM format, the backhand loop/drive against topspin balls exhibited significant differences across all zones, particularly in the backhand zone, where RRM (46.8%) and LLM formats (51.2%) showed a pronounced disparity.

When juxtaposing RLM and LLM formats, the backhand flip showed a significant difference in the forehand zone, with the RLM format at 19.7% and the LLM format at 12.4%. Lastly, in the examination of the LRM versus the LLM format, the short push was significantly more prevalent in the backhand zone in the LLM format (12.9%) than in the LRM format (9.3%). The backhand loop/drive against topspin balls also showed significant differences in the backhand zone, with the LRM format at 28.6% and the LLM format at 51.2%.

## Discussion

This study sought to explore and evaluate the effectiveness of strokes that indirectly contributed to both scoring and conceding points across the four major formats of table tennis competition. The research revealed notable differences in the impact of stroke techniques and ball placement strategies within these formats. These differences were particularly pronounced for what we define as the 'winner-assisting stroke' and the 'momentum-diminishing stroke', shedding light on their distinct roles in rally outcomes.

### Winner-Assisting Stroke

As for the antepenultimate stroke in the hitting sequence, its execution is crucial and indirectly contributes to a player's ability to score. Ten strategic stroke actions were pivotal for crafting decisive shots across various competition formats. The serve remains the only stroke in table tennis that is not subject to restrictions, offering players a unique chance to control the game's flow (Gomez, 2017; Zhang et al., 2007). The results showed that during rallies completed within RRM match formats, players often aimed to minimize their opponents' attacking chances by serving side-spin balls to the middle zone, compelling them to return from less advantageous positions. This middle zone had emerged as a particularly beneficial target area, with its frequency of use in matches on the rise (Wang, 2019), potentially due to its ability to disrupt the opponent's rhythm, thus securing an advantage for the server on the third shot (Malagoli et al., 2014). Conversely, left-handed players might favor targeting the opponent's forehand area for creating following attacks, a strategy possibly informed by their frequent encounters with right-handed players, which enhanced their understanding of how to capitalize on the forehand area. Research by Malagoli Lanzoni et al. (2019) suggested that left-handed players possessed a strategic edge in table tennis due to their relative rarity, which can catch opponents off guard. This could explain why left-handed players were particularly effective at exploiting their opponents' weaknesses in the LRM format. Furthermore, Loffing et al.'s (2009) research in tennis indicates that left-handed players may hold an advantage, as their play patterns are less familiar to their rivals. This "left-handed advantage" might similarly manifest in table tennis, particularly during serve and receive phases.

A notable distinction was observed in the application of the forehand flip and the backhand flip aiming at the forehand zone during RRM and LLM formats. This pattern hinted that right-handed players, when competing against counterparts with a similar grip, often favored the forehand flip. By doing so, they introduced greater spin and diversity to the ball's trajectory, thereby seizing the upper hand in the match. The adoption of this tactic aligns with Djokic's findings from 2002, which indicated that right-handed players frequently employed forehand techniques to regulate the game's tempo and heighten their opponents' unease. Conversely, left-handed players appeared to have a preference for the backhand flip, potentially due to their greater experience in initiating attacks with this stroke when pitted against right-handed adversaries. This inclination was supported by Malagoli Lanzoni et al. (2019)'s research, which revealed a higher frequency of backhand techniques among left-handed players. This preference may stem from their deeper acquaintance and comfort with backhand maneuvers (Malagoli Lanzoni et al., 2014).

The backhand loops or drives against a backspin ball are prevalent offensive maneuvers in table tennis, as noted by various studies (Fuchs and Lames, 2021; Gomez et al., 2017; Qu et al., 2010). Analysis of match data reveals that targeting the backhand area was particularly beneficial in rallies performed within the RRM and LLM formats, whereas in the RLM format, the forehand area proved to be more potent. In both RRM and LLM formats, compelling opponents to respond with their backhand substantially raised the likelihood of securing a critical follow-up shot. Conversely, in the RLM format, directing the ball to the middle zone effectively constrained opponents from executing large-angle diagonal returns, thereby opening up scoring opportunities to both flanks. Furthermore, the short push stroke was less frequently employed to place the ball to the backhand zone during RRM matches. This tendency may suggest that right-handed players, when confronting opponents who share the same grip style, tend to shy away from driving the ball towards their opponent's backhand area, likely to prevent the opponent from capitalizing on potential attacking chances.

### Momentum-Diminishing Stroke

Considering the penultimate stroke in the hitting sequence, it plays a pivotal role in creating difficulties for the conceding player. Eleven specific stroke actions were intricately connected to the nuanced outcomes of indirectly conceding points across diverse competitive formats. The forehand and backhand flicks, along with loops and drives against the topspin, constitute the foundational offensive mechanisms that are vital for building a favorable momentum (Fuchs and Lames, 2021; Yu and Gao, 2022). However, our study findings suggest that employing the backhand flip to direct the ball towards an opponent's backhand side in a rally within the RRM format was fraught with risks, and this risk was amplified when returning the ball to the opponent's forehand side in the LLM format as opposed to the RLM format. Regarding the forehand flip technique, a marked disparity existed in the balls that resulted in point losses between RRM and RLM formats, with the majority of points lost in the RRM format occurring on the forehand side, whereas in the RLM format, the backhand side was more susceptible. To navigate these scenarios effectively, players should heighten their focus on executing straight-line flicks with their forehand, thereby introducing an unexpected dimension to their game.

Secondly, in the context of forehand loops/drives against non-topspin balls, there was a tendency to aim for the opponent's forehand and backhand areas during rallies in the RRM format. In contrast, players in the RLM format predominantly focused on backhand offensive plays, which, due to their lack of variation, could heighten the risk of conceding points. This divergence may stem from the technical attributes and tactical inclinations associated with different grip styles. As Djokic et al. (2020) highlighted in their research, the choice of a grip significantly influences a player's stroke techniques and strategic decisions. When confronting non-topspin balls, the decision to opt for backhand or forehand loops/drives warranted careful contemplation. The results underscore the fact that launching an attack was not invariably synonymous with achieving a beneficial result.

When comparing rallies from the RRM with the LRM format, it was observed that the long push was employed more often in the LRM format. Deployed as an unexpected tactic, the long push can catch opponents off balance, focusing on compressing the timing and spatial aspects of the stroke to elicit errors or subpar returns from rivals (Yu and Gao, 2022). However, the data indicated that the forehand area was disproportionately responsible for point losses, likely owing to the inadequate quality of forehand shots which opponents could readily counter with forceful attacks. Furthermore, considering backhand loops and drives against non-topspin balls, RRM players tended to target both the forehand and backhand areas of their opponents, whereas LRM players more evenly dispersed their attack targets. This variance might stem from greater reliance on the stability and control afforded by the backhand area, with players opting to aim for the middle area to decrease the chances of losing points. In contrast between the LRM and LLM formats, the short push was notably more prevalent in the backhand area during the LLM format. This could suggest that left-handed players, when competing against right-handed opponents, were more inclined to leverage the backhand techniques of their dominant hand to dictate the game's tempo.

Lastly, when examining the differences between RLM and LRM formats, significant variations were noted in the application of backhand loops and drives on topspin balls targeting both the forehand and backhand areas. LRM players, when executing backhand loops and drives to the opponent's forehand area, tended to lose more points, whereas in the RLM format, the backhand area was where more points were conceded. This could imply that such tactical disparities were linked to players' adaptability to their opponents' grip styles and strategic in-game decisions (Djokic, 2003). Considering the increasing scoring rates observed during the stalemate phase (Wang et al., 2022; Zhang et al., 2018), professional players should utilize these insights to refine their strategies when facing opponents with different dominant hand preferences, thus avoiding falling into a disadvantageous position.

The current study is of paramount importance to athletes, coaches, trainers, and physiotherapists, as it may contribute significantly to shaping training and competitive strategies. It highlights the significant impact of stroke techniques on scoring, providing athletes with a clear roadmap for skill enhancement and equipping coaches with a solid foundation for strategic planning. Athletes could fine-tune their strategies, especially during crucial moments, to maximize scoring opportunities and minimize errors when facing opponents with varying handedness. Coaches could effectively guide athletes in choosing the optimal stroke and shot placement by leveraging their own strengths and exploiting the weaknesses of their opponents, particularly those with different grip styles. Physical trainers should focus on helping athletes develop wrist and arm strength, as well as agility, to execute key strokes that could significantly increase their scoring potential. The technical maneuvers discussed in the study require a high level of physical fitness and skill from athletes, and the overuse of certain muscle groups can lead to sports injuries. Consequently, physiotherapists should concentrate on preventing injuries to the wrists, shoulders, back, and knees to ensure proper technique and reduce the risk of injury. By applying these findings, the sports community can gain a deeper understanding of the demands of competition and the physical condition of athletes, ultimately enhancing competitive performance and the outcomes of matches.

The groundbreaking contribution of this study lies in its thorough analysis of the impact of stroke techniques and corresponding ball landing locations on indirect scoring and point concession across the four predominant table tennis match formats. However, the study is not without its limitations, and it should be noted that our findings are specific to matches between male players only and may not be universally applied. Furthermore, the three-zone partition method, while allowing for an estimation of the ball's depth based on the stroke technique used to execute a shot, falls short in capturing the nuanced differences that can arise from applying the same stroke technique to the same target zone. For example, when executing a short push to the middle zone, the ball's landing point can vary significantly, ranging from being very close to the net to somewhere in between the net and the table's end line. Despite these constraints, the study offers innovative insights, serving as a valuable resource for enhancing competitive table tennis performance.

## Conclusions

In this study, we conducted a meticulous analysis and comparison of the performance of various strokes, examining both technique and ball placement, to understand their indirect effects on scoring and conceding points across the four primary formats of table tennis competition.

For strokes that assist in winning points, our analysis indicated that the tactical placement of the ball during critical moments, such as executing a normal sidespin serve, backspin/no-spin serve, short push, forehand flip, backhand flip, and backhand loop/drive against both topspin and non-topspin balls, significantly impacted the success of rallies. The influence of these strokes was observed to vary considerably among the different match formats.

On the other hand, strokes that led to losing momentum were identified by the areas where the ball landed during the backspin/no-spin serve, short and long pushes, and the aforementioned flips and loop/drives against topspin and non-topspin balls. These strokes were found to distinctly contribute to the negative outcomes of rallies, with noticeable variations among the four match formats.

We advise professionals, including coaches, athletes, analysts, and others, to utilize these findings to enhance their practical strategies and to refine their training regimens. By doing so, they can optimize their competitive edge and achieve peak performance in table tennis matches.
